# Quality indicators to ensure excellence in glaucoma care: the GlauCCare Spanish consensus

**DOI:** 10.1136/bmjophth-2024-002078

**Published:** 2025-05-30

**Authors:** Francisco J Munoz-Negrete, Julian Garcia-Feijoo, Elena Millá, Luis Pablo-Júlvez, Javier A Urcola, Cristina Camunas-Sevilla, Félix Rubial-Bernárdez, María C Rodríguez-Vázquez

**Affiliations:** 1 Ophthalmology, Hospital Universitario Ramón y Cajal, Madrid, Spain; 2 Ophthalmology, Hospital Clínico Universitario San Carlos, Madrid, Spain; 3 Ophthalmology, Hospital Clinic de Barcelona, Barcelona, Spain; 4 Ophthalmology, Hospital Universitario Miguel Servet, Zaragoza, Spain; 5 Ophthalmology, Hospital Universitario de Álava, Vitoria, Spain; 6 Quality, Hospital Universitario de Toledo, Toledo, Spain; 7 Área Sanitaria de Lugo, A Mariña e Monforte de Lemos, Lugo, Spain; 8 Market Access, Santen Pharmaceutical Spain S.L, Madrid, Spain

**Keywords:** Glaucoma

## Abstract

**Topic:**

To develop quality indicators to be included for the certification of excellence care in glaucoma units in Spain.

**Clinical relevance:**

The certificate of excellence in care in glaucoma units is expected to enhance clinical outcomes and patient satisfaction, but also to optimise the use of resources and promote an efficient and effective patient care system.

**Methods:**

The Delphi methodology was used to obtain a consensus on quality indicators in glaucoma care. The scientific committee and an advisory group created a 182-item questionnaire classified by indicator type: care structure, care process and outcomes. A two-round Delphi survey was conducted among a panel of expert ophthalmologists in Spain, and a 9-point Likert-type scale was used for data analysis.

**Results:**

After the two rounds, 39 panellists reached consensus on 166 out of 182 items (91.2%). By indicator type, consensus was reached on 38 (88.4%) indicators related to care structure, on 85 (87.6%) indicators related to care process and on all indicators 42 (100%) related to outcomes.

**Conclusion:**

This consensus identified a set of quality indicators that will help to develop the certification of excellence in glaucoma care units. This certification will facilitate best clinical practices and better health outcomes for glaucoma patients. Limitations of the study include the consensus nature of results, potential bias from the lengthy questionnaire and the focus on experts only in Spain, limiting generalisability.

**WHAT IS ALREADY KNOWN ON THIS TOPIC**

- Glaucoma is a progressive burdensome eye disease with no standardised processes or quality indicators in glaucoma care. The definition and development of quality indicators might allow a better assessment of the quality of care for an optimal approach that results in maximal benefits for the patient.

**WHAT THIS STUDY ADDS**

- This Delphi consensus provides the expert view on what the standards of quality of care should be to help harmonise care, reduce unwarranted variations in treatment and ensure the greatest benefit for the glaucoma patient.

**HOW THIS STUDY MIGHT AFFECT RESEARCH, PRACTICE OR POLICY**

- Based on the agreed indicators, it will be possible to work on a standardised norm endorsed by the Spanish Glaucoma Society (SEG) that will guarantee excellence in glaucoma care in centres that demonstrate compliance, paving the way towards homogeneous glaucoma care that provides the best services and outcomes for patients.

## Introduction

 Glaucoma is the main cause of irreversible vision loss worldwide and the second most common cause of irreversible vision impairment.^1^ It has an estimated global prevalence of 3.5% in people aged 40–80 years,^2^ leading to moderate or severe visual impairment in 4.1 million people and blindness in 3.6 million people.^3^ Given that glaucoma prevalence increases with age^4^ and the rise of population ageing, it is estimated that the number of people affected by glaucoma will reach 111.8 million by 2040.^5^

The severe consequences of vision loss include both physical and mental health-related problems, and a significant impact on the quality of life.^6 7^ From a societal perspective, adults with vision loss may experience lower employment rates and increased healthcare resource use, which translates into high healthcare burden and economic impact.^8 9^

According to the WHO, the implementation of tools to assess the provision of eye care services is crucial in the prevention of blindness.^8^ For instance, tools for the assessment of glaucoma services/units to ensure excellent quality of care.^8^

Traditionally, the quality of care has been determined through quality indicators, which are measurable items used to supervise the quality of provided care. Quality indicators may be classified into three categories: (1) indicators related to the care structure, concerning human, material or facility resources that guarantee an adequate care structure to provide the best patient care; (2) indicators related to the care process, that is, related to the protocols and methodologies followed to guarantee an adequate care process to provide the best patient care and (3) indicators related to the outcomes, that is, representing a specific outcome or derived from specific actions taken throughout the care process that ensure the best health outcomes for patients.^10^

Quality indicators for ensuring care quality have been developed in Spain for other specialties, such as psoriasis units,^11^ integral care units for patients with inflammatory bowel disease^12^ and rheumatology day hospital units,^13^ among others. Specifically in the field of glaucoma, Castejón-Cervero *et al* assessed the compliance with European Glaucoma Society (EGS) recommendations on diagnosis and treatment of glaucoma in Spain, by using the Achievable Benchmarks of Care approach.^14^ Nevertheless, no specific studies or recommendations for measuring the quality of care in glaucoma units have been published in Spain so far. Recently, Iorio-Aranha *et al* developed quality indicators to assess glaucoma care in Portugal; however, their results cannot be directly extrapolated to other countries with different healthcare systems.^15^

The aim of this project was to develop clear quality standards to help harmonise care, reduce unwarranted variations in treatment and ensure the greatest benefit for the patient. In the present study, the Delphi methodology was adopted to reach a consensus on the quality indicators that should be included for the certification of glaucoma units to ensure excellence in care. The Delphi method consists of a prospective research technique with proven reliability, whose purpose is to evaluate the degree of consensus or visualise the points of discrepancy between experts on the research topic.

## Materials and methods

### Study design

In the GlauCCare consensus, the Delphi methodology was used to obtain a consensus on quality indicators in glaucoma care among a panel of expert ophthalmologists. The Delphi methodology consists of a structured process of collecting opinions through two rounds of expert consultation, with the aim of analysing the level of agreement on the appropriateness of the proposed indicators.

**Figure 1:**
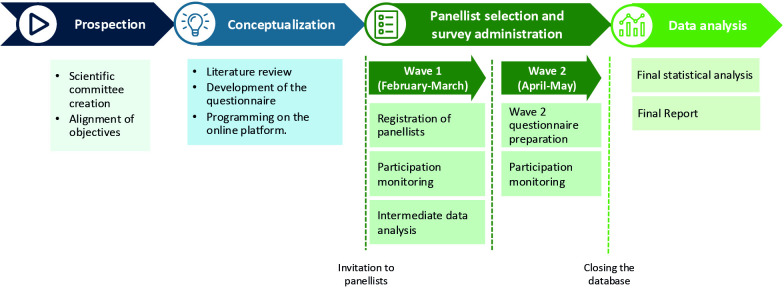
Steps followed in the GlauCCare Spanish consensus.

This project was carried out in several successive phases: (1) prospection (creation of the scientific committee (SC), alignment of objectives); (2) conceptualisation (literature review, questionnaire development, programming on the online platform); (3) panellist selection and survey administration (two waves) and (4) statistical data analysis of collected data (figure 1).

The SC included five experts in glaucoma management in Spanish reference hospitals, who belong to glaucoma scientific societies and have authored a large volume of publications. Based on the literature review and clinical expertise, the SC designed the questionnaire for the Delphi survey.

### Questionnaire design

The questionnaire was designed from a multidisciplinary perspective, developed by an SC of five expert ophthalmologists and reviewed by an advisory group consisting of a healthcare quality expert, a pharmacist and a healthcare manager.

The final questionnaire, created ad-hoc for this project, included 182 items and was structured in five sections: (1) optimisation of the care process (49 items); (2) prevention and early detection of glaucoma (22 items); (3) diagnosis of glaucoma (26 items); (4) glaucoma treatment (50 items); (5) glaucoma follow-up (35 items). Sections 1–5 were also classified per indicator type, namely (a) indicator of care structure, (b) indicator of care process and (c) indicator of outcomes. An additional section to characterise the participating panellists and their usual clinical practice was also included (15 items). Sections 1–5 were rated using a 9-position Likert-type scale: 1–3 (completely disagree, not important at all or not confident at all), 4–6 (neutral) and 7–9 (completely agree, extremely important or completely confident).

The panellist’s characterisation section included descriptive questions, with different answer modalities (single choice, multiple choice, open field).

### Panellist selection and survey administration

Ophthalmologists with expertise in glaucoma and members of the Spanish Glaucoma Society (SEG) from different regions of Spain were invited to participate. All the experts who agreed to participate had to meet the selection criteria. Those criteria to ensure sufficient experience of the panellists were established: (1) ophthalmologists should be involved in the management and treatment of glaucoma; (2) ophthalmologists should belong to a glaucoma unit/section; (3) they should have at least 5 years of experience in glaucoma; (4) they should see at least 30 glaucoma patients per week and (5) they should perform at least 40 glaucoma surgeries per year. The questionnaire administration took place between 1 February 2024 and 19 March 2024 (wave 1) and between 17 April 2024 and 8 May 2024 (wave 2).

### Data analysis

For data analysis of the Likert-type scale questions, answers were systematised in three proposed levels: 1–3, 4–6 and 7–9, and the percentage of panellists scoring in each level was calculated for each item. Consensus was defined when 70% or more of the panellists were aligned in their answer: 1–3 (consensus in disagreement), 4–6 (neutral) and 7–9 (consensus in agreement).^16^ Those items for which consensus was not reached in the first wave were reconsulted in a second wave.

Nominal type variables were described by means of frequency (number and percentage), and continuous variables by central tendency and dispersion measurement.

It was determined whether there were significant differences between the responses obtained in both waves using the Boker test. The significance level established was 0.05 bilateral.

## Results

### Panellist participation

**Figure 2:**
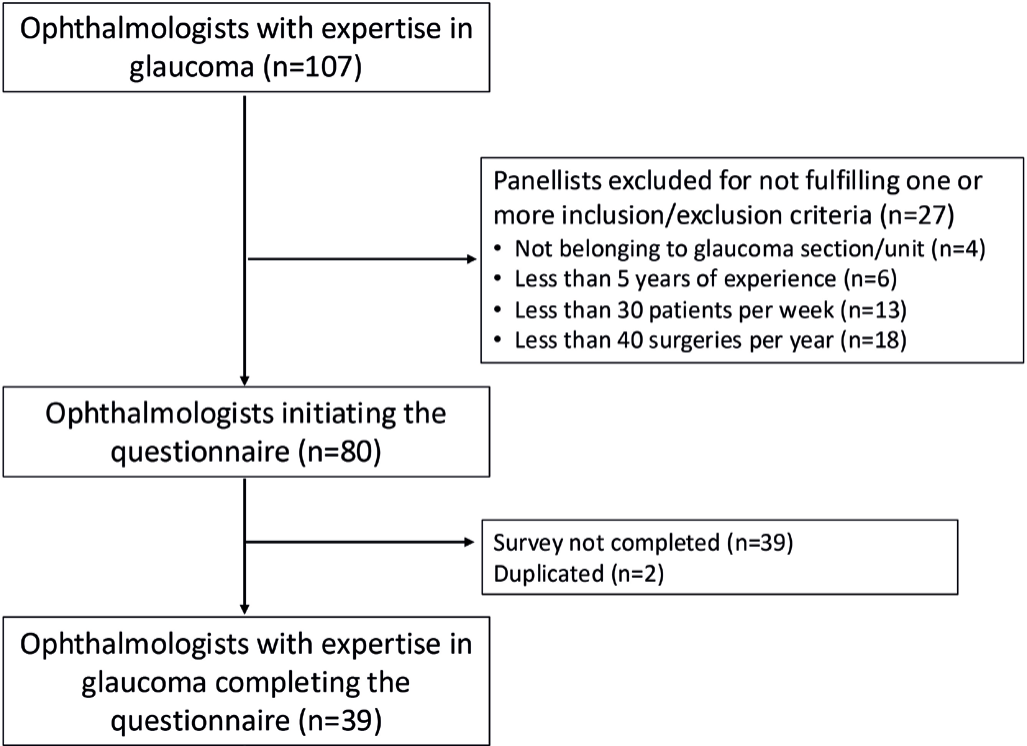
Selection of panellists.

A total of 107 ophthalmologists with expertise in glaucoma from all over Spain were invited to participate. The survey was highly rigorous and specific to ensure that the knowledge of the country’s leading glaucoma experts was gathered. Among all the invited ophthalmologists, 27 were discarded, so 80 panellists met the inclusion/exclusion criteria and were candidates to complete the questionnaire, but due to the time required to answer, they did not finish answering the questionnaire. Finally, a total of 39 ophthalmologists completed the two rounds of the Delphi survey (figure 2). It is important to note that there is no established consensus regarding the optimal number of participants in a Delphi study. The critical factors in this methodology are the careful selection of potential expert participants, the strategies implemented for their recruitment and the measures taken to ensure a high response rate from those experts who are instrumental in the evaluation and decision-making processes related to the subject under investigation. Consequently, population representation is not a requisite in this methodology.

### Panellist profile

The participating panellists were men (59.0%) and women (41.0%) aged 51.9±8.4 years, mainly working in tertiary centres (94.8%), both in the public and private sectors. The sample was geographically balanced. Panellists reported a wide expertise: 69% had more than 20 years of experience and 33.3% had between 11 and 20 years of experience. In addition, 82.1% of panellists reported attending >30 patients per week and 53.8% performed >100 glaucoma surgeries per year. In online supplemental table 1, a full description of the panellists can be found.

In their clinical practice, 64.1% of panellists reported having >75% of patients with high intraocular pressure (IOP) and who were older than 60 years. A full description of the profile of the patients seen by the panellists through the different phases of the care process (diagnosis, treatment and follow-up) can be found in online supplemental table 2.

### Overall results

At the end of the Delphi process, consensus was reached on 166 out of 182 items (165 on ‘agreement’ and 1 on ‘disagreement’), meaning that panellists agreed with 90.7% of the proposed indicators. Five items were not raised in wave 2 due to redundancies with other items on which consensus was reached in wave 1 (online supplemental table 3). A total of 14 items were raised in wave 2, 3 of which reached consensus and the 11 remaining items of which did not reach consensus, either in the first or in the second wave (online supplemental table 3, items without consensus highlighted in red).

### Indicators related to the care structure

Of the 182 proposed indicators, 43 were considered indicators related to the care structure. Consensus on agreement was reached on 38 (88.4%) indicators, all in wave 1. The remaining 5 indicators were re-evaluated in wave 2, but no consensus was reached for any of these (online supplemental table 3).

For care process optimisation, having glaucoma-trained medical staff (indicating that any health professional involved in the management of a patient with glaucoma must be trained) in all specialty centres and hospitals was considered to be a care structure indicator by 97.4% of panellists (table 1). According to the experts, indicators of care structure for glaucoma treatment include having highly trained medical personnel with extensive experience in surgical procedures (100%), sufficient operating theatres (97.4%), available clean rooms for other procedures (97.4%), traceability systems in case devices (indicating to have traceability systems for the operation and updating of medical devices) (97.4%) and to promote attendance at congresses to be updated on glaucoma (97.4%) (table 1). Finally, to carry out a good follow-up, 97.4% of panellists considered that having all the equipment and instruments and proper access to them are indicators of care structure (table 1).

**Table 1:** Indicators of care structure with a degree of consensus on agreement >97%

Questionnaire section	Indicator	Agree (7–9) (%)
Optimisation of the care process	Have glaucoma-trained medical staff in all specialty centres and hospitals	97.4
Glaucoma treatment	Have highly/specialised trained medical personnel with extensive experience in the performance of surgical procedures	100.0
Glaucoma treatment	Have sufficient operating theatres to ensure that patients receive the intervention they need in a timely manner	97.4
Glaucoma treatment	Have clean rooms available for procedures that do not require the use of an operating theatre (eg, administration of intravitreal therapies, intracameral therapies, post-surgical check-ups, etc)	97.4
Glaucoma treatment	Have traceability systems in case devices are used	97.4
Glaucoma treatment	Promote attendance at national and international symposia and congresses to learn about the latest advances in the field of glaucoma	97.4
Glaucoma follow-up	Have all the instruments necessary to be able to carry out a good follow-up	97.4
Glaucoma follow-up	Have all the equipment necessary to be able to carry out a good follow-up	97.4
Glaucoma follow-up	Have access to instruments/equipment specifically for patient follow-up	97.4

### Indicators related to the care process

Of the 182 proposed indicators, 97 were considered indicators related to the care process. Of these 97 indicators, consensus on agreement was reached on 85 (87.6%): 83 in wave 1, and 2 in wave 2. Consensus on disagreement was reached on 1 indicator in wave 2 (online supplemental table 3).

Regarding indicators for care process optimisation, 97.4% of panellists agreed that patients should be informed about treatment and prognosis at all times (table 2). In addition, having the results of the screening monitored by a specialised ophthalmologist was considered an indicator for prevention and early detection of glaucoma by 97.4% of panellists (table 2). According to the experts, indicators for the care process in glaucoma treatment include individualised change of treatment in the event of non-response (97.4%), to carry out an individual assessment when surgical intervention is considered (97.4%), ensuring that the type of surgery is adapted to each patient (97.4%) and having a pre-, intra- and post-surgery checklist (97.4%). Finally, regular training updates for all staff were agreed by 100% of panellists (table 2). To carry out the follow-up, panellists considered that performing tonometry (100.0%) and fundus examination (without dilating the pupil, 97.4%) at all visits were quality indicators of care process (table 2).

**Table 2:** Indicators of care process with a degree of consensus on agreement >97%

Questionnaire section	Indicator	Agree (7–9) (%)
Optimisation of the care process	Keep the patient informed at all times about the progress and results of his or her treatment, as well as the medium/long-term prognosis	97.4
Prevention and early detection of glaucoma	Have the results of the screening monitored by a specialised ophthalmologist	97.4
Diagnosis of glaucoma	Protocolise progression studies to ensure their application and reliability, in order to simplify decision making	97.4
Glaucoma treatment	Conduct regular training updates for all staff to ensure they are aware of the latest developments	100.0
Glaucoma treatment	In the event of non-response to initial treatment, change the therapeutic group based on the characteristics of each patient	97.4
Glaucoma treatment	Carry out an individual and holistic assessment in those cases in which surgical intervention is considered due to risk of progression, despite correct compliance with pharmacological therapy	97.4
Glaucoma treatment	Ensure that the choice of the type of surgery is adapted to the needs of each patient	97.4
Glaucoma treatment	Have a surgical checklist (pre-, intra- and post-surgery)	97.4
Glaucoma follow-up	Perform tonometry at all visits	100.0
Glaucoma follow-up	Perform fundus examination at all visits (without dilating the pupil)	97.4

### Indicators related to outcomes

Of the 182 proposed indicators, 42 were considered indicators related to outcomes. Consensus on agreement was reached on all items (100%) in the first wave. The complete list of all indicators related to outcomes can be found in online supplemental table 3.

Regarding indicators related to outcomes, 94.9% of panellists agreed on ensuring that all patients are provided with care in a reasonable time to obtain the greatest benefit, and a high percentage of patients are satisfied with the care received and the course of their disease (table 3).

**Table 3:** Indicators related to outcomes with a degree of consensus on agreement >94%

Questionnaire section	Indicator	Agree (7–9) (%)
Optimisation of the care process	Ensure that all patients are provided with care in a reasonable time to obtain the greatest benefit	94.9
Optimisation of the care process	Ensure that a high percentage of patients are satisfied with the care received	94.9
Optimisation of the care process	Ensure that a high percentage of patients are satisfied with the course of their disease	94.9
Diagnosis of glaucoma	Ensure that a high percentage of patients receive a diagnosis of glaucoma before they have vision impairment/loss	97.4
Glaucoma treatment	Simplify pharmacological treatment as far as possible to maximise benefits	97.4
Glaucoma treatment	Minimise the percentage of patients with complex treatment regimens	97.4
Glaucoma treatment	Ensure that a high percentage of patients undergo surgery in an optimal timeframe	94.9
Glaucoma treatment	Assess the use of advanced/penetrating interventions by analysing the risk/benefit balance on an individual patient basis	94.9
Glaucoma follow-up	Ensure that a high percentage of patients are accountable for their care and adherent to treatment	94.9

For glaucoma diagnosis-related indicators, 97.4% of panellists agreed on ensuring that a high percentage of patients receive a diagnosis of glaucoma before they have vision impairment/loss (table 3). Regarding indicators related to outcomes on glaucoma treatment, simplifying pharmacological treatment (97.4%), minimising the percentage of patients with complex treatment regimens (94.9%), ensuring that most patients undergo surgery in an optimal timeframe (94.9%) and individually assessing the use of advanced/penetrating interventions (94.9%), were considered by the vast majority of panellists (table 3). Among the indicators related to outcomes on glaucoma follow-up, 94.9% of panellists agreed on ensuring that a high percentage of patients are accountable for their care and adherent to treatment (table 3).

## Discussion

This study presents a consensus on recommendations for quality indicators considered indispensable for guaranteeing excellent quality of care in glaucoma units. The indicators for evaluating the performance of the glaucoma units in Spain identified in the present study are in line with the research priorities for glaucoma care pointed out by the EGS.^17^

While Spanish Ophthalmology Services provide specialised and dedicated care in glaucoma units, there is still a need to standardise care across different glaucoma units. It is crucial to establish standardised quality indicators, not only to harmonise care, but also to improve the quality of services. Standardised quality indicators may eventually promote best practices, improve clinical care and ensure better health outcomes and patient satisfaction. Therefore, the findings of the present study can empower glaucoma units to further improve the quality of care and can ensure that all patients receive maximum benefit.

This study found that those indicators related to glaucoma treatment in care structure achieved the highest level of consensus. Although this section of the questionnaire was the most extensive, five of the nine indicators with the highest level of consensus were under this section. This emphasises the importance of prioritising the patient’s well-being and quality of life while adopting a patient-centred approach. In this line, there was high consensus on several indicators related to patient involvement, such as shared decision-making, assessment of their perspective or improvement with patient communication/training. This is consistent with recently published data demonstrating the benefits of adequate patient training,^18^ or the need to develop specific tools for the evaluation of the patient’s perspective, which has become a key point in clinical trials.^19^

The importance of appropriate and sustainable disease management from a public system point of view is also highlighted. Glaucoma is a chronic and progressive disease, with associated costs derived from treatments, disease progression and side effects. Therefore, optimising the use of resources and promoting an efficient and effective patient care system is fundamental. In fact, a high degree of consensus was achieved on the need for resources, such as medical and administrative staff, equipment and facilities. Moreover, the importance of having highly trained staff with extensive training and experience in surgical procedures was also noted. There was unanimity on the importance of staff training to keep abreast of the latest advances, suggesting that having highly specialised staff guarantees a quality care process. Additionally, the study emphasised the need for constant updating, for both staff and patients by training, and promoting innovation through attendance at congresses or symposia or participation in clinical trials. Finally, the implementation of technologies such as telemedicine and remote patient care was considered to be important, which has adopted an important role in medicine and ophthalmology, particularly since the COVID-19 pandemic. Glaucoma care may benefit from remote eye examinations (tonometry, perimetry and fundus imaging), while it may also allow for a more patient-centred and accessible future.^20^

However, there was a lack of consensus on several indicators related to multidisciplinary coordination. This suggests that those phases of the care process with greater involvement of primary care always require active collaboration with the ophthalmology department.

Most of the statements assessed in the ‘care process’ category were related to standardising care; including both tests or procedures required at each stage of the care process, or the need for clear criteria and specific protocols. This is in line with the latest Spanish clinical practice guidelines for advanced glaucoma, which emphasise the importance of protocolising care.^21^ Quality standards with unanimity in the care process section include performing tonometry at all follow-up visits. Given that the reduction of IOP is the only effective measure to slow glaucoma progression, tonometry examinations are strongly recommended in the follow-up.^2^ Moreover, IOP can be treated to prevent further vision deterioration with either pharmacological treatment (eye drops), laser, surgery or a combination of these.^22^ In contrast, no agreement was reached on the usefulness of performing genetic studies for glaucoma diagnosis, even in those with glaucoma with a high hereditary component. It may be too early to consider these indicators as indispensable for ensuring quality of care, since the future of glaucoma research is aimed at personalising treatment based on genetic profile or exploring potential gene therapies.^23^

Finally, in the present study, all indicators related to ‘outcomes’ were agreed in the first wave. This suggests that experts considered it very relevant to prioritise those procedures or treatments with the least impact on the patient’s life; thus, assigning great importance to the best health outcomes for patients.

To the best of our knowledge, few specific studies on the use of quality indicators to assess the performance of a glaucoma unit have been published so far. Two guidelines on quality standards for glaucoma in adults^24 25^ and one summary of Process Indicators for Measuring Quality of Care in Glaucoma have been previously issued.^26^ The first nationwide study on quality indicators to assess glaucoma care was recently developed in Portugal.^15^ It reported a set of 30 quality indicators for measuring the performance of glaucoma units. Nevertheless, their results cannot be directly translated to other settings with different cultural, social and healthcare systems. In contrast with the quality indicators identified by Iorio-Aranha to assess glaucoma care in Portugal,^15^ the present study incorporates not only structure/process and outcome indicators, but also extensively considers each of the phases of the care process, being a more comprehensive investigation. In Spain, Castejón-Cervero *et al* extracted quality and process-of-care indicators from the EGS guidelines on diagnosis and treatment of glaucoma in Spain.^14^ Pending the development of standards of quality in the field of Glaucoma, here we present a prospection for further developing appropriate indicators for evaluating the performance of glaucoma units in Spain.

### Future directions

Having identified potential indicators that guarantee excellence in quality of care, it is now necessary to define the standard for certifying glaucoma units. Therefore, future steps of the GlauCCare project should be to qualify and validate the proposed quality indicators to achieve a useful and reliable tool for glaucoma unit excellence validation. Finally, and endorsed by the SEG, a certification standard for glaucoma units should be prepared. Since quality indicators include a range of variables from human resources, patient care and support, accessibility to management (detection, referral, treatment and follow-up), translating this diversity into a validated tool is a challenging undertaking.

### Strengths and limitations

The main strength of the study is the broad consensus reached among panellists, with percentages higher than 97%, virtually reaching unanimity. Furthermore, the participation of glaucoma experts from across Spain integrated knowledge of a wide panel of experts while also ensuring geographical representativeness. Another strength of the study is intrinsic to the nature of the Delphi methodology: it allows anonymity between participants with controlled feedback provided in a structured manner. However, the study has some limitations that need to be acknowledged. First, the results of the study reflect the consensus opinion, as per intrinsic characteristics of Delphi methodology. The questionnaire length (182 items) may also be another limitation, because tiredness-induced bias cannot be ruled out. Finally, the study only included experts practising in Spain, and the results may not be extrapolated to other countries.

## Conclusions

This study has identified a set of quality indicators that will allow excellent care in glaucoma units to be ensured. This is expected to enhance clinical outcomes and patient satisfaction, but also to optimise the use of resources and promote an efficient and effective patient care system.

With the identified quality indicators, a regulation for the subsequent certification of excellence in glaucoma care units will be developed. Thanks to this certification, best clinical practices and better health outcomes for glaucoma patients might be achieved.

## Supplementary material

10.1136/bmjophth-2024-002078 online supplemental table 1

10.1136/bmjophth-2024-002078 online supplemental table 2

10.1136/bmjophth-2024-002078 online supplemental table 3

## Data Availability

All data relevant to the study are included in the article or uploaded as supplementary information.

## References

[1] Steinmetz JD, Bourne RRA, Briant PS (2021). Causes of blindness and vision impairment in 2020 and trends over 30 years, and prevalence of avoidable blindness in relation to VISION 2020: the Right to Sight: an analysis for the Global Burden of Disease Study. Lancet Glob Health.

[2] Kang JM, Tanna AP (2021). Glaucoma. Med Clin North Am.

[3] Burton MJ, Ramke J, Marques AP (2021). The Lancet Global Health Commission on Global Eye Health: vision beyond 2020. Lancet Glob Health.

[4] McMonnies CW (2017). Glaucoma history and risk factors. J Optom.

[5] Tham YC, Li X, Wong TY (2014). Global prevalence of glaucoma and projections of glaucoma burden through 2040: a systematic review and meta-analysis. Ophthalmology.

[6] Quaranta L, Riva I, Gerardi C (2016). Quality of Life in Glaucoma: A Review of the Literature. Adv Ther.

[7] Moussavi S, Chatterji S, Verdes E (2007). Depression, chronic diseases, and decrements in health: results from the World Health Surveys. Lancet.

[8] World Health Organization (WHO) Blindness and vision impairment. https://www.who.int/news-room/fact-sheets/detail/blindness-and-visual-impairment.

[9] Darbà J, Marsà A (2022). Ambulatory and hospital care of glaucoma in Spain and associated medical costs. J Med Econ.

[10] Donabedian A (1988). The Quality of Care: How Can It Be Assessed. JAMA.

[11] Salgado-Boquete L, Arias-Santiago S, Belinchón-Romero I (2023). [Translated article] Selection of Quality Indicators for the Certification of Psoriasis Units: The CUDERMA Project Delphi Consensus Study. Actas Dermo-Sifiliográficas.

[12] Grupo Español de Trabajo en Enfermedad de Crohn y Colitis Ulcerosa (GETECCU) (2016). Normalización de los indicadores de calidad para unidades de atención integral a pacientes con enfermedad inflamatoria intestinal.

[13] Sociedad Española de Reumatología (SER) Estándares e indicadores de calidad de las unidades de hospital de día en reumatología.

[14] Castejón-Cervero MA, Jiménez-Parras R, Fernandez-Arias I (2011). Evaluation of compliance with the EGS guidelines in Spain, using Achievable Benchmarks of Care (ABC®) methodology: the IMCA Study. Eur J Ophthalmol.

[15] Iorio-Aranha F, de Freitas C, Rocha-Sousa A (2024). Nationwide consensus on quality indicators to assess glaucoma care: A modified Delphi approach. Eur J Ophthalmol.

[16] Sullivan GM, Artino AR (2013). Analyzing and interpreting data from likert-type scales. J Grad Med Educ.

[17] Azuara-Blanco A, McCorry N, Tatham AJ (2024). European Glaucoma Society research priorities for glaucoma care. *Br J Ophthalmol*.

[18] Sng JJ, Ang BCH, Hoo WCS (2023). The Effectiveness of a Nurse-led Glaucoma Education on Patient Knowledge and Compliance Motivation Levels: A 1-year Prospective Case Series. J Curr Glaucoma Pract.

[19] Vinokurtseva A, Quinn MP, Wai M (2023). Evaluating Measurement Properties of Patient-Reported Outcome Measures in Glaucoma: A Systematic Review. Ophthalmol Glaucoma.

[20] Jo JJ, Pasquale LR (2024). Recent developments of telemedicine in glaucoma. Curr Opin Ophthalmol.

[21] Díez-Álvarez L, Beltrán-Agullo L, Loscos J (2023). Advanced glaucoma. Clinical practice guideline. Arch Soc Esp Oftalmol (Engl Ed).

[22] Mohan N, Chakrabarti A, Nazm N (2022). Newer advances in medical management of glaucoma. Indian J Ophthalmol.

[23] Jayaram H, Kolko M, Friedman DS (2023). Glaucoma: now and beyond. Lancet.

[24] National Institute for Health and Care Excellence (NICE) Glaucoma: quality standard.

[25] Health Quality Ontario Glaucoma: care for adults (quality standards).

[26] Iorio-Aranha F, Peleteiro B, Rocha-Sousa A (2021). A Scoping Review of Process Indicators for Measuring Quality of Care in Glaucoma. J Glaucoma.

