# Supplementary material for: Quality indicators to ensure excellence in glaucoma care: the GlauCCare Spanish consensus
Source: BMJ Open Ophthalmol. 2025 May 30;10(1):e002078. doi: 10.1136/bmjophth-2024-002078 (PMC12128410; doi:10.1136/bmjophth-2024-002078)
Supplement: online supplemental table 1 [file bmjophth-10-1-s001.pdf]

Supplementary Table 1. Characteristics of participant experts.

	N=39
**Gender**, n (%)	
Female	16 (41.0%)
Male	23 (59.0%)
**Age** (years), mean (SD)	51.9(8.4)
**Geographical distribution**, n (%)	
Andalusia	8 (20.5%)
Asturias	1 (2.6%)
Basque country	1 (2.6%)
Canary Islands	1 (2.6%)
Cantabria	1 (2.6%)
Castile and Leon	3 (7.7%)
Castile-La Mancha	2 (5.1%)
Catalonia	6 (15.4%)
Community of Madrid	7 (17.9%)
Galicia	2 (5.1%)
La Rioja	1 (2.6%)
Navarre	1 (2.6%)
Region of Murcia	1 (2.6%)
Valencia	4 (10.3)
**Centre of work**, n (%)	
Primary care	1 (2.6%)
Secondary care hospital	1 (2.6%)
Tertiary hospital	37 (94.8%)
**Years of experience**, n (%)	
5-10	3 (7.7%)
11-20	13 (33.3%)
>20	23 (59.0%)
**Visited patients (per week)**, n (%)	
30-49	7 (17.9%)
>50	32 (82.1%)
**Glaucoma surgeries performed (per year)**, n (%)	
40-49	3 (7.7%)
50-99	15 (38.5%)
>100	21 (53.8%)

SD: standard deviation
