# Supplementary material for: Quality indicators to ensure excellence in glaucoma care: the GlauCCare Spanish consensus
Source: BMJ Open Ophthalmol. 2025 May 30;10(1):e002078. doi: 10.1136/bmjophth-2024-002078 (PMC12128410; doi:10.1136/bmjophth-2024-002078)
Supplement: online supplemental table 2 [file bmjophth-10-1-s002.pdf]

Supplementary Table 2. Characteristics of patients attended by participant experts.

	Percentage of panellists with the following percentage of patients with the stated characteristics					
	**<10%**	**10-25%**	**25-50%**	**50-75%**	**75-90%**	**>90%**
**Clinical characteristics**						
Patients with high IOP	5.1%	0.0%	2. 6%	28.2%	46.2%	17.9%
Patients > 60 years	0.0%	0.0%	5.1%	30.8%	58.9%	5.1%
Patients with ophthalmological pathologies (other than myopia)	2. 6%	28.2%	43.6%	17.9%	5.1%	2. 6%
Patients < 60 years	7.7%	48.7%	41.0%	2.6%	0.0%	0.0%
Black patients	84.6%	12.8%	2.6%	0.0%	0.0%	0.0%
Patients on corticoids	48.7%	38.5%	12.8%	0.0%	0.0%	0.0%
Patients with diabetes	10.3%	56.4%	15.4%	17.9%	0.0%	0.0%
Patients with myopia	10.3%	35.9%	41.0%	10.3%	2.6%	0.0%
**Diagnostic characteristics**						
Patients diagnosed after suffering irreversible vision loss	48.7%	33.3%	7.7%	7.7%	2.6%	0.0%
Patients identified through screening in primary care and subsequent diagnosis in the ophthalmology department.	56.4%	25.6%	10.3%	2.6%	5.1%	0.0%
Patients identified in follow-up consultations in the ophthalmology department	0.0%	17.9%	25.6%	20.5%	25.6%	10.3%
Patients diagnosed in time to receive treatment	2.6%	10.3%	17.9%	15.4%	30.8%	23.1%
**Treatment-related characteristics**						
Patients whose treatment includes laser intervention	38.5%	46.2%	10.3%	5.1%	0.0%	0.0%
Patients not controlled with any of the available treatment options	76.9%	12.8%	7.7%	0.0%	2.6%	0.0%
Patients whose treatment includes surgery	7.7%	30.8%	33.3%	25.6%	2.6%	0.0%
Patients controlled with pharmacological therapy alone	2.6%	5.1%	35.9%	35.9%	15.4%	5.1%

Patients receiving timely and appropriate treatment	2.6%	7.7%	17.9%	28.2%	28.2%	15.4%
**Follow-up characteristics**						
Patients meeting their therapeutic goals (achieving the expected health outcomes)	0.0%	2.6%	10.3%	53.8%	30.8%	2.6%
Patients adhering to treatment	0.0%	2.6%	23.1%	51.3%	12.8%	10.3%
Patients with adequate follow-up	0.0%	0.0%	15.4%	23.1%	51.3%	10.3%

IOP: intraocular pressure
