# Supplementary material for: Quality indicators to ensure excellence in glaucoma care: the GlauCCare Spanish consensus
Source: BMJ Open Ophthalmol. 2025 May 30;10(1):e002078. doi: 10.1136/bmjophth-2024-002078 (PMC12128410; doi:10.1136/bmjophth-2024-002078)
Supplement: online supplemental table 3 [file bmjophth-10-1-s003.pdf]

Supplementary table 3. Summary of the results of the Delphi study.

Questionnaire section	Questionnaire sub-section	Indicator	Type of indicator (A= Care structure, B=Care process, C=outcomes)	Final results			Comments
				No agreement	Neutral	Totally in agreement	
A. Optimisation of the care process	A.1. Necessary human resources	Provide primary care (PC) medical centres with medical staff trained in glaucoma.	A	15.4%	30.8%	53.8%	Consensus not reached
		Have glaucoma-trained medical staff in all speciality centres and hospitals.	A	0.0%	2.6%	97.4%	
		Have qualified professionals (optometrists, nurses, etc.) specifically trained in glaucoma.	A	5.1%	15.4%	79.5%	
		Have a training protocol, so as to guarantee the correct training of the professionals involved in glaucoma management.	B	2.6%	10.3%	87.2%	
		Have adequate staffing to provide care to all patients in an appropriate time frame.	A	2.6%	2.6%	94.9%	
		Have administrative personnel specifically trained to organize appointments based on previously established prioritization criteria (face-to-face and telematic).	A	5.1%	17.9%	76.9%	
		Have a case manager to evaluate those cases in which patients come to the Glaucoma Unit for consultation and/or resolution of doubts.	A	10.3%	17.9%	71.8%	
	A.2. Resource optimization and accessibility	Availability of a sufficient number of ophthalmology consultation modules to provide care to all patients in a reasonable time in outpatient centers.	A	2.6%	7.7%	89.7%	
		Have a minimum number of consultation modules dedicated specifically to glaucoma based on demand (monographic consultations).	A	2.6%	2.6%	94.9%	
		Periodically review the waiting lists to update them based on established prioritization criteria (benefit, severity).	B	0.0%	7.7%	92.3%	
		Have total flexibility for the realization of interventions.	B	2.6%	5.1%	92.3%	
		Ensure that all patients are provided with care in a reasonable time to obtain the greatest benefit.	C	5.1%	0.0%	94.9%	
		Value blended learning in those cases in which it guarantees an optimization of the assistance process.	B	10.3%	17.9%	71.8%	
		Have sufficient personnel and resources for the optimal implementation of telemedicine in the context of glaucoma.	A	10.3%	17.9%	71.8%	
		Periodically evaluate the distribution and accessibility of different glaucoma-related services.	B	7.7%	12.8%	79.5%	
		Ensure efficient mobility in the care environment, which facilitates the process for patients and makes efficient use of time and resources.	C	7.7%	15.4%	76.9%	
		Provide space in the Glaucoma Unit for activities with patient associations and/or training activities for patients.	A	10.3%	17.9%	71.8%	
	A.3. Patient care and support	Establish effective communication with patients and their relatives, fostering a relationship of trust in which they can express their experiences.	B	0.0%	5.1%	94.9%	
		Periodically carry out studies that analyze patients' perception of their health status (PROMs), in order to identify areas for improvement.	B	0.0%	12.8%	87.2%	
		Periodically conduct studies that analyze the experience and satisfaction of patients with the medical care received (PREMs), in order to identify areas for improvement.	B	2.6%	10.3%	87.2%	
		Evaluate patient experience at each step of the care process in terms of accessibility to different resources.	B	0.0%	23.1%	76.9%	
		Ensure that a high percentage of patients are satisfied with the care received.	C	0.0%	5.1%	94.9%	
		Ensure that a high percentage of patients are satisfied with the health education they have received.	C	0.0%	12.8%	87.2%	
		Ensure that a high percentage of patients are satisfied with the course of their disease.	C	0.0%	5.1%	94.9%	
		Develop a patient support program that lists available resources that can be made available to patients.	B	2.6%	20.5%	76.9%	
		Offer patients at all times access to psychosocial support to cope with the diagnosis and prognosis of their pathology.	B	5.1%	20.5%	74.4%	
		Develop face-to-face training programs for patients (and their relatives/caregivers), adapted to different profiles.	B	7.7%	20.5%	71.8%	
		Develop online training programs for patients (and their relatives/caregivers), adapted to different profiles.	B	5.1%	12.8%	82.1%	
		Provide patients with tailored deliverables with complete and simple information regarding their disease and treatment(s).	B	2.6%	7.7%	89.7%	
		Have qualified nursing staff/nursing assistants available to train patients regarding their treatment (posology, treatment administration technique, control of adverse effects, post-intervention care, etc.).	A	7.7%	12.8%	79.5%	
		Facilitate tools and resources for patients to actively monitor and manage their disease (e.g. intraocular pressure registry, treatment compliance registry, etc.).	B	5.1%	12.8%	82.1%	
		Promote participation in informative and/or training activities developed in collaboration with patient associations.	C	7.7%	15.4%	76.9%	
		Inform patients in detail about therapeutic alternatives, their risks and benefits, so that they can participate in informed decision making.	B	0.0%	5.1%	94.9%	
		Make alternative communication channels available to the patients/legals in case they have questions regarding their pathology/treatment.	B	2.6%	5.1%	92.3%	
		Keep the patient informed at all times about the progress and results of his or her treatment, as well as the medium/long-term prognosis.	B	0.0%	2.6%	97.4%	
		Guarantee the protection and correct flow of patient data throughout the care process.	B	0.0%	5.1%	94.9%	
		Ensure that the patient participates in joint decision making and therapeutic goal setting.	C	0.0%	7.7%	92.3%	
		Ensure that a high percentage of patients have contact information and access to the unit.	C	0.0%	7.7%	92.3%	
	A.4. Greater optimization of the care process	Ensure compliance with the guidelines established in national and European guidelines.	C	0.0%	7.7%	92.3%	
		Have specific itineraries and protocols for each ophthalmologic pathology, including glaucoma, based on available clinical guidelines.	B	0.0%	5.1%	94.9%	
		Audit all the results obtained in the approach to patients, regardless of the type of intervention (self-audit or third-party audit).	B	2.6%	15.4%	82.1%	
		Conduct regular multidisciplinary clinical sessions in which the approach is presented and the most complex/controversial cases are discussed.	B	2.6%	5.1%	92.3%	
		Have a registry of patients treated in the Glaucoma Unit.	A	0.0%	5.1%	94.9%	
		Have a registry of complex patients treated in the Glaucoma Unit.	A	0.0%	5.1%	94.9%	
		Have communication and information systems between levels of care.	A	5.1%	5.1%	89.7%	
		Ensure high access to teleconsultations in patients with low complexity glaucoma.	C	7.7%	10.3%	82.1%	
		Ensure that a high percentage of glaucoma patients under follow-up report improvement and/or maintain their quality of life.	C	2.6%	7.7%	89.7%	
		Ensure that a high percentage of patients do not have vision impairment in the past year.	C	0.0%	12.8%	87.2%	
		Ensure that a high percentage of patients benefit from follow-up in single visits/high-resolution consultations.	C	0.0%	10.3%	89.7%	

Questionnaire section	Questionnaire sub-section	Indicator	Type of indicator (A= Care structure, B=Care process, C=outcomes)	Final results			Comments
				No agreement	Neutral	Totally in agreement	
B. Prevention and early detection of glaucoma	B.1. Early detection from PC	Have a specific protocol for glaucoma screening.	B	2.6%	7.7%	89.7%	
		Promote population screening campaigns.	C	2.6%	20.5%	76.9%	
		Participate in glaucoma awareness campaigns.	C	2.6%	12.8%	84.6%	
		For a correct screening, a complete anamnesis should be performed on any patient over 40 years of age to identify a patient with possible glaucoma.	B	5.1%	23.1%	71.8%	
		For a correct screening, a complete anamnesis should be performed on relatives of patients diagnosed with glaucoma.	B	5.1%	17.9%	76.9%	
		For a correct screening, a complete anamnesis of patients at risk should be carried out to identify a patient with possible glaucoma.	B	5.1%	12.8%	82.1%	
		Have PC physicians specifically trained in the identification of patients at high risk for glaucoma.	A	2.6%	25.6%	71.8%	
		Have other professionals (nurses, optometrists, opticians, etc.) specifically trained in the identification of patients at high risk of glaucoma.	A	2.6%	17.9%	79.5%	
		Have the possibility of certain diagnostic tests performed by PC physicians, with the necessary supervision by the ophthalmologist.	B	5.1%	20.5%	74.4%	
		Have efficient methods of visualising complementary tests in the PC consultation.	B	15.4%	28.2%	56.4%	Consensus not reached
		Availability of an optotype table in PC consultations.	A	2.6%	25.6%	71.8%	
		Availability of a non-mydriatic retinograph in PC offices.	A	7.7%	10.3%	82.1%	
		Availability of a tonometer in PC consultations.	A	7.7%	23.1%	69.2%	Consensus not reached
		Ensure that professionals performing intraocular pressure measurements are aware of and able to assess the possible influence that the state of the cornea may have on the onset of glaucoma.	B	5.1%	15.4%	79.5%	
		Have the results of the screening monitored by a specialised ophthalmologist.	B	2.6%	0.0%	97.4%	
		Availability of resources for “satellite consultations” in which the PC physician performs the screening tests and the ophthalmologist interprets them.	A	10.3%	7.7%	82.1%	
	B.2. Referral to ophthalmology	Have a specific protocol in the PC center for the referral of patients with possible glaucoma to the ophthalmology service.	B	2.6%	15.4%	82.1%	
		Ensure referral of all patients with suspected glaucoma.	C	2.6%	7.7%	89.7%	
	B.3. Optimization of early detection	Have an agile and fluid communication channel between PC and specialized care.	A	5.1%	2.6%	92.3%	
		Extend diabetic retinopathy screening to glaucoma screening with the resources available in PC.	B	2.6%	10.3%	87.2%	
		In those patients with risk factors in whom glaucoma is not identified, repeat screening in PC will be considered at a time defined by individual patient characteristics.	B	5.1%	15.4%	79.5%	Consensus reached in wave 2
		Ensure that a high percentage of patients attend regular screening examinations.	C	5.1%	7.7%	87.2%	
C. Diagnosis of glaucoma	C.1. Necessary tests	Include in the diagnosis a complete anamnesis, fundus examination, tonometry, campimetry, gonioscopy and pachymetry.	B	5.1%	10.3%	84.6%	
		Include in the diagnosis a complete anamnesis, fundus examination, tonometry, campimetry, gonioscopy, pachymetry and retinography.	B	7.7%	7.7%	84.6%	
		Include in the diagnosis a complete anamnesis, fundus examination, tonometry, campimetry, gonioscopy, pachymetry and OCT.	B	5.1%	7.7%	87.2%	
		Include in the diagnosis a complete anamnesis, fundus examination, tonometry, campimetry, gonioscopy, pachymetry, retinography and OCT.	B	5.1%	7.7%	87.2%	
		Include angio-OCT as a necessary test to establish a correct diagnosis of glaucoma.	B	79.5%	15.4%	5.1%	Consensus (in disagreement) reached in wave 2
		Include anterior segment OCT as a necessary test to establish a correct diagnosis of glaucoma.	B	56.4%	38.5%	5.1%	Consensus not reached
		Include a complete genetic study in patients < 40 years of age, syndromic or with glaucoma with a high hereditary component as a necessary test to establish a correct diagnosis of glaucoma.	B	35.9%	46.2%	17.9%	Consensus not reached
	C.2. Equipment and methodology	Have all the instruments necessary to obtain a good diagnosis.	A	2.6%	5.1%	92.3%	
		Have all the necessary equipment to obtain a good diagnosis.	A	5.1%	5.1%	89.7%	
		Availability of high-resolution diagnostic equipment to facilitate early diagnosis and proper patient management.	A	5.1%	5.1%	89.7%	
		Availability of a portable non-mydriatic retinograph for early diagnosis in PC centers.	A	10.3%	15.4%	74.4%	
		Availability of portable OCT to enable early diagnosis in PC centres.	A	51.3%	23.1%	25.6%	Consensus not reached
		Availability of a portable tonometer to enable early diagnosis in PC centres.	A	25.6%	12.8%	61.5%	Consensus not reached
		Periodic calibration of such equipment to ensure correct readings.	B	0.0%	5.1%	94.9%	
		Perform tonometry using the Goldmann applanation technique, with a single measurem	B	17.9%	43.6%	38.5%	Consensus not reached. This item was not raised in Waves 2, as consensus was reached on the indicator which qualifies that several measures should be carried out
		Perform tonometry using the Goldmann flattening technique, taking several measurements at different times of the day for greater diagnostic accuracy.	B	10.3%	12.8%	76.9%	
		Availability of other tonometry techniques (reformulated in wave 2 as: Availability of alternative techniques/tonometers for performing tonometry tests).	A	23.1%	23.1%	53.8%	Consensus not reached
		Protocolise progression studies to ensure their application and reliability, in order to simplify decision making.	B	0.0%	2.6%	97.4%	
		Promote the use of artificial intelligence based on imaging tests to optimize the diagnostic process and reduce the workload of specialists.	B	7.7%	20.5%	71.8%	
	C.3. Diagnostic optimization	Perform diagnostic tests in a reasonable time to obtain a diagnosis for all patients as soon as possible.	C	5.1%	5.1%	89.7%	
		Have a protocol establishing the diagnostic process, with the required tests and the personnel in charge of them.	B	0.0%	10.3%	89.7%	
		Have rapid strategies for performing perimetry and/or campimetry, which allow to shorten the scanning time and increase the number of patients scanned.	B	0.0%	10.3%	89.7%	
		Ensure that patients who receive a diagnosis at the first consultation receive a treatment regimen on the same day (high-resolution consultation).	C	0.0%	10.3%	89.7%	
		Have an ophthalmologist in charge of monitoring the results of all diagnostic tests.	A	5.1%	15.4%	79.5%	
		Ensure that a high percentage of patients receive diagnostic confirmation within an adequate waiting time.	C	0.0%	10.3%	89.7%	
		Ensure that a high percentage of patients receive a diagnosis of glaucoma before they have vision impairment/loss.	C	0.0%	2.6%	97.4%	

Questionnaire section	Questionnaire sub-section	Indicator	Type of indicator (A= Care structure, B=Care process, C=outcomes)	Final results			Comments
				No agreement	Neutral	Totally in agreement	
D. Glaucoma treatment	D.1. Improvement of pharmacological treatment	Ensure compliance with the guidelines established in national and European guidelines.	C	2.6%	7.7%	89.7%	
		To have a protocol establishing the most appropriate criteria and steps of pharmacological treatment for all patients (single protocol).	B	20.5%	28.2%	51.3%	Consensus not reached. This item was not raised in Waves 2, as consensus was reached on the indicator which qualifies that protocols should be available for each patient profile for each patient profile
		Have a protocol that establishes the most appropriate pharmacological treatment criteria and steps for each patient profile.	B	2.6%	7.7%	89.7%	
		Assess the estimated degree of compliance for each therapeutic option according to the patient's characteristics.	C	2.6%	5.1%	92.3%	
		Ensure that pharmacological treatment is chosen after a global assessment of the patient, including criteria such as ocular surface condition, systemic pathology, etc.	B	2.6%	2.6%	94.9%	
		Evaluate the condition of the ocular surface prior to prescribing a topical treatment.	B	0.0%	5.1%	94.9%	
		In the event of non-response to initial treatment, change the therapeutic group based on the characteristics of each patient.	B	0.0%	2.6%	97.4%	
		Stagger treatment by adding drugs from another therapeutic group in all patients who do not reach the intraocular pressure target.	B	5.1%	7.7%	87.2%	
		Ensure that, in combined treatments, the posology (dosage and administration interval) is the same for all treatments to help obtain better adherence.	B	0.0%	12.8%	87.2%	
		Simplify pharmacological treatment as far as possible to maximise benefits.	C	0.0%	2.6%	97.4%	
		Ensure that a high percentage of patients receive treatment as a fixed-dose combination (FDC).	C	5.1%	12.8%	82.1%	
		Minimise the percentage of patients with complex treatment regimens.	C	2.6%	0.0%	97.4%	
		Evaluate therapeutic strategies in each phase, favoring the use of fixed combinations when feasible, in order to reduce the number of packages as much as possible and improve adherence.	B	2.6%	2.6%	94.9%	
		Assess the possibility of carrying out pharmacogenetic tests prior to prescribing treatment.	B	41.0%	33.3%	25.6%	Consensus not reached
		Availability of tables containing the active ingredients and trade names of the different drugs, allowing easy identification of therapeutic equivalents in case of need.	B	2.6%	17.9%	79.5%	
		Have a pharmacovigilance protocol in place.	B	5.1%	30.8%	64.1%	Consensus not reached
	D.2. Improvement of trabeculoplasty treatment	Ensure compliance with the guidelines established in national and European guidelines	C	0.0%	15.4%	84.6%	
		Have a protocol that establishes the criteria for recommending this laser intervention.	B	2.6%	10.3%	87.2%	
		Have up-to-date, quality laser equipment that is properly calibrated and maintained to ensure treatment efficacy and safety.	A	0.0%	10.3%	89.7%	
		Have highly trained medical personnel with extensive experience in performing these laser interventions.	A	0.0%	7.7%	92.3%	
	D.3. Improvement of surgical treatment	Ensure compliance with the guidelines established in national and European guidelines.	C	0.0%	10.3%	89.7%	
		Have a protocol that establishes the criteria for recommending surgical intervention.	B	0.0%	15.4%	84.6%	
		Carry out an individual and holistic assessment in those cases in which surgical intervention is considered due to risk of progression, despite correct compliance with pharmacological therapy.	B	0.0%	2.6%	97.4%	
		Ensure adequate training of all members of the unit in all glaucoma surgery techniques, from trabeculectomy to minimally invasive or penetrating techniques.	B	0.0%	7.7%	92.3%	
		Ensure that the choice of the type of surgery is adapted to the needs of each patient.	B	0.0%	2.6%	97.4%	
		Involve, as much as possible, the patient in decision making.	B	0.0%	5.1%	94.9%	
		Opt for minimally invasive surgeries in those cases where indicated.	C	2.6%	5.1%	92.3%	
		Opt for minimally penetrating surgeries in those cases where indicated.	C	2.6%	5.1%	92.3%	
		Evaluate for each case the possible association of antimetabolites to achieve higher success rates.	B	0.0%	5.1%	94.9%	
		Evaluate for each case the implantation of drainage implants.	B	0.0%	5.1%	94.9%	
		Have a detailed protocol specifying the pre-anesthetic tests required for each type of surgery.	B	2.6%	5.1%	92.3%	
		Have a detailed protocol establishing the selection criteria for each anesthetic technique.	B	2.6%	15.4%	82.1%	
		Prioritize the use of topical anesthesia whenever possible.	C	7.7%	12.8%	79.5%	
		Have sufficient operating theatres to ensure that patients receive the intervention they need in a timely manner.	A	0.0%	2.6%	97.4%	
		Have clean rooms available for procedures that do not require the use of an operating theatre (e.g. administration of intravitreal therapies, intracameral therapies, post-surgical check-ups, etc.).	A	0.0%	2.6%	97.4%	
		Have highly trained medical personnel with extensive experience in the performance of surgical procedures.	A	0.0%	0.0%	100.0%	
		Have sufficient resources available to ensure an adequate average waiting list time.	A	2.6%	2.6%	94.9%	
		Opt for combined surgeries in all patients with glaucoma who require cataract surgery and whose clinical situation makes it advisable.	C	2.6%	10.3%	87.2%	
		Ensure that a high percentage of patients undergo surgery in an optimal timeframe.	C	0.0%	5.1%	94.9%	
		Audit the results obtained in surgical interventions (self-audit or third-party audit).	C	0.0%	12.8%	87.2%	
		Have traceability systems in case devices are used.	A	2.6%	0.0%	97.4%	
		Have a surgical checklist (pre-intra- and post-surgery).	B	2.6%	0.0%	97.4%	
		New indicator (included in OLA2) - Have a system of alerts in the operating room that collects all incidents that occur during an operation, whether or not they involve the patient.	B	7.7%	15.4%	76.9%	
	D.4. Promote innovation	Assess the implementation of new pharmacological treatments, analyzing the risk/benefit balance individually for each patient.	C	2.6%	5.1%	92.3%	
		Assess the use of advanced/penetrating interventions by analysing the risk/benefit balance on an individual patient basis.	C	0.0%	5.1%	94.9%	
		Promote attendance at national and international symposia and congresses to learn about the latest advances in the field of glaucoma.	A	0.0%	2.6%	97.4%	
		Conduct regular training updates for all staff to ensure they are aware of the latest developments.	B	0.0%	0.0%	100.0%	
		Participate in novel clinical trials to contribute to advances in glaucoma treatment.	A	0.0%	5.1%	94.9%	
		Participate in novel research in the treatment of glaucoma.	A	0.0%	5.1%	94.9%	
		Publish/communicate in prestigious journals or forums of interest.	C	7.7%	2.6%	89.7%	

Questionnaire section	Questionnaire sub-section	Indicator	Type of indicator (A= Care structure, B=Care process, C=outcomes)	Final results			Comments
				No agreement	Neutral	Totally in agreement	
E. Glaucoma follow-up	E.1. Necessary tests	Include fundus examination, tonometry, campimetry, gonioscopy and pachymetry in the follow-up.	B	12.8%	33.3%	53.8%	Consensus not reached. This item was not reconsidered in Waves 2, as consensus was reached on other indicators that expand the minimum tests.
		Include fundus examination, tonometry, campimetry, gonioscopy, pachymetry and retinography in the follow-up.	B	7.7%	23.1%	69.2%	Consensus not reached
		Include fundus examination, tonometry, campimetry, gonioscopy, pachymetry and OCT in the follow-up.	B	5.1%	2.6%	92.3%	
		Include fundus examination, tonometry, campimetry, gonioscopy, pachymetry, retinography and OCT in the follow-up.	B	5.1%	10.3%	84.6%	
		Perform tonometry at all visits.	B	0.0%	0.0%	100.0%	
		Perform fundus examination at all visits (without dilating the pupil).	B	2.6%	0.0%	97.4%	
		Perform dilated fundus examination periodically (not every visit) depending on the patient's characteristics.	B	5.1%	17.9%	76.9%	
		Perform fundus examination with dilated pupil whenever change of status is suspected (in cases of surgery).	B	2.6%	10.3%	87.2%	
		Perform gonioscopy periodically (not every visit) depending on patient characteristics.	B	10.3%	10.3%	79.5%	
		Perform gonioscopy whenever a change of status is suspected.	B	2.6%	5.1%	92.3%	
		Perform gonioscopy before performing any surgical procedure.	B	0.0%	5.1%	94.9%	
		Perform postoperative gonioscopy in all patients.	B	12.8%	10.3%	76.9%	Consensus reached in wave 2
		Obtain papilla images periodically (not every visit) depending on the patient's characteristics.	B	2.6%	12.8%	84.6%	
		Obtain papilla images whenever status change is suspected.	B	0.0%	7.7%	92.3%	
		Perform campimetry on all visits.	B	25.6%	43.6%	30.8%	Consensus not reached. This item was not raised in Waves 2, as consensus was reached on the indicator that states that campimetry should be performed periodically.
		Perform campimetry periodically (not every visit) depending on the patient's characteristics.	B	2.6%	2.6%	94.9%	
		Perform OCT at all visits.	B	17.9%	25.6%	56.4%	Consensus not reached. This item was not raised in Waves 2, as consensus was reached on the indicator that qualifies that OCT should be performed periodically.
		Perform OCT periodically (not every visit) depending on patient characteristics.	B	7.7%	12.8%	79.5%	
		Have all the instruments necessary to be able to carry out a good follow-up.	A	0.0%	2.6%	97.4%	
		Have all the equipment necessary to be able to carry out a good follow-up.	A	0.0%	2.6%	97.4%	
		Have access to instruments/equipment specifically for patient follow-up.	A	0.0%	2.6%	97.4%	
		Have a system for alerting patients of delays/non-attendance at periodic check-ups.	A	0.0%	7.7%	92.3%	
	E.2. Tracking optimization	Ensure compliance with the guidelines described in the main clinical practice guidelines, both national and European.	C	0.0%	10.3%	89.7%	
		Have a protocol that establishes how patients with glaucoma should be followed up.	B	0.0%	17.9%	82.1%	
		Have a protocol that establishes the tests to be performed in follow-up visits, as well as their frequency depending on the patient's characteristics.	B	0.0%	7.7%	92.3%	
		Have a protocol that establishes the professional who should be in charge of each consultation.	B	2.6%	17.9%	79.5%	
		Have a protocol that establishes in which cases direct consultation with the specialist should be requested.	B	2.6%	7.7%	89.7%	
		Have a protocol with specific instructions for assessing adherence to treatment.	B	5.1%	15.4%	79.5%	
		Have a protocol that includes the criteria for requesting consultation according to the most frequent comorbidities.	B	2.6%	17.9%	79.5%	
		Establish therapeutic objectives and specific compliance times for each patient, reviewed periodically.	C	0.0%	7.7%	92.3%	
		In case of non-compliance with therapeutic objectives within the established time frame, review each case individually in order to adjust the treatment.	B	5.1%	7.7%	87.2%	
		In case of non-compliance with therapeutic objectives in a large percentage of patients, convene multidisciplinary meetings to review the protocols.	B	2.6%	15.4%	82.1%	
		Ensure that a high percentage of patients are accountable for their care and adherent to treatment.	C	0.0%	5.1%	94.9%	
		Minimize the percentage of patients who do not attend follow-up visits.	C	2.6%	5.1%	92.3%	
		Ensure a minimum percentage of patients achieve adequate control of their glaucoma.	C	2.6%	5.1%	92.3%	
